# Integrally calcified solitary fibrous tumor in the retroperitoneum: a case report and review of the literature

**DOI:** 10.1186/s40792-016-0143-8

**Published:** 2016-02-13

**Authors:** Takehiro Maki, Syotaro Fujino, Kenjiro Misu, Hiroyuki Kaneko, Hitoshi Inomata, Makoto Omi, Masatoshi Tateno, Kazuyoshi Nihei

**Affiliations:** Department of Surgery, Kushiro Red Cross Hospital, 21-14, Shineichyo, Kushiro, Hokkaido 085-8512 Japan; Department of Pathology, Kushiro Red Cross Hospital, 21-14, Shineichyo, Kushiro, Hokkaido 085-8512 Japan

**Keywords:** Solitary fibrous tumor, Retroperitoneum, Calcification

## Abstract

Solitary fibrous tumor (SFT) is a rare stromal neoplasm and usually occurs in the thoracic cavity. We here report a case of retroperitoneal SFT with prominent calcification. A 64-year-old man presented with an incidentally detected retroperitoneal mass in the right upper abdomen. Imaging tests indicated an integrally calcified mass. The lesion was observed for 2 years and laparoscopically resected according to the patient’s wish. Microscopically, the mass was mostly occupied by calcification and proliferous spindle cells were scattered with positive CD34 expression. We diagnosed morphologically benign SFT and the patient remained disease-free 1 year after the excision. There has been no report of such integrally calcified SFT. Retroperitoneal SFT is difficult to make a preoperative diagnosis, and careful follow-up after the excision is recommended because morphological malignancy does not always correspond to clinical malignancy.

## Background

Solitary fibrous tumor (SFT) is known as an uncommon mesenchymal neoplasm. It mainly develops in thoracic cavity but extrathoracic SFTs have been also reported. We here present a rare case of retroperitoneal SFT with marked calcification, followed by a review of the literature.

## Case presentation

**Fig. 1:**
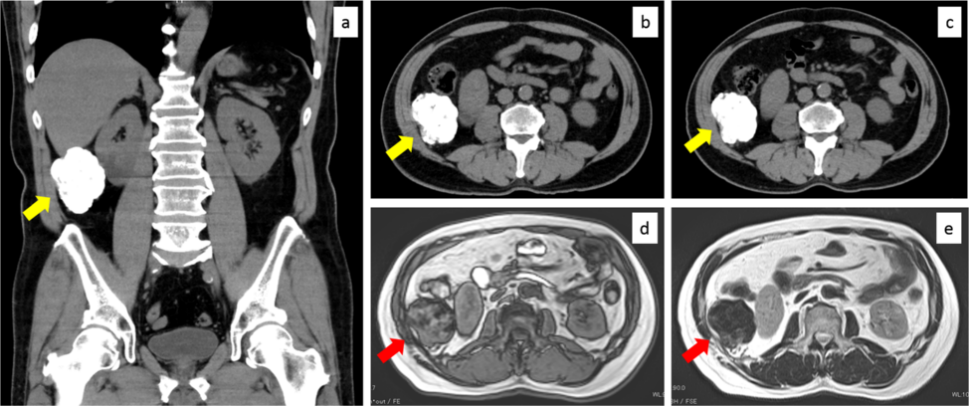
Preoperative images. **a**, **b** Images of plain computed tomography on the patient’s first visit. A mass with extremely high densities was located next to the undersurface of the right hepatic lobe and the right kidney (*yellow arrows*). It was 6.5 cm in the longest diameter. **c** An image of plain computed tomography 2 years after the patient’s first visit (*yellow arrow*). There were no remarkable changes of the mass in its appearance or size. **d**, **e** Magnetic resonance imaging on the patient’s first visit. The mass showed low signal intensities on both **d** T1- and **e** T2-weighted images (*red arrows*)

A 64-year-old Japanese man without any symptoms had a medical checkup, and abdominal ultrasonography revealed a mass of the right upper abdomen with acoustic shadow. He had hypertension and underwent appendectomy for appendicitis by McBurney’s incision at 13 years old. He was 162 cm tall and weighted 70 kg (body mass index, 26.8). His blood pressure, pulse, oxygen saturation, and body temperature were 146/76 mmHg, 66 beats/min, 97 %, and 36.2 °C, respectively. His abdomen was flat and soft without any palpable masses. Laboratory examinations revealed no remarkable abnormalities of complete blood count, inflammation, liver function, renal function, electrolytes, or coagulation system. Plain computed tomography showed a mass with extremely high densities; it was 6.5 cm in the longest diameter and located next to the undersurface of the right hepatic lobe and the right kidney (Fig. 1a, b). Contrast enhanced computed tomography did not reveal any other lesions in the chest and abdomen. Magnetic resonance imaging showed low signal intensities on both T1- and T2-weighted images (Fig. 1d, e). We considered that the lesion mainly consisted of calcification and diagnosed large peritoneal pearl body or sponge-induced granuloma due to the past appendectomy. Malignant fibrous histiocytoma, dedifferentiated liposarcoma, or extraskeletal osteosarcoma were considered as differential diagnoses although they were atypical or rare according to the imaging findings. We informed the patient about a low possibility of malignancy of the lesion and he wished observation. Two years after, the patient hoped surgical excision although the lesion showed a stable imaging in computed tomography (Fig. 1c).

**Fig. 2:**
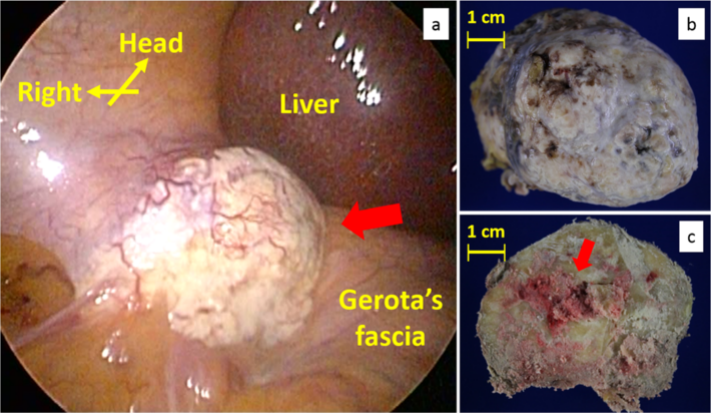
A laparoscopic image of the right upper abdomen and photographs of the excised specimen. **a** In the right upper retroperitoneum, a white and hard mass was observed next to the right hepatic lobe and the right kidney. **b** The excised specimen presented calcified mass. **c** Division surface of the specimen presented mainly calcification and partially red soft tissues

**Fig. 3:**
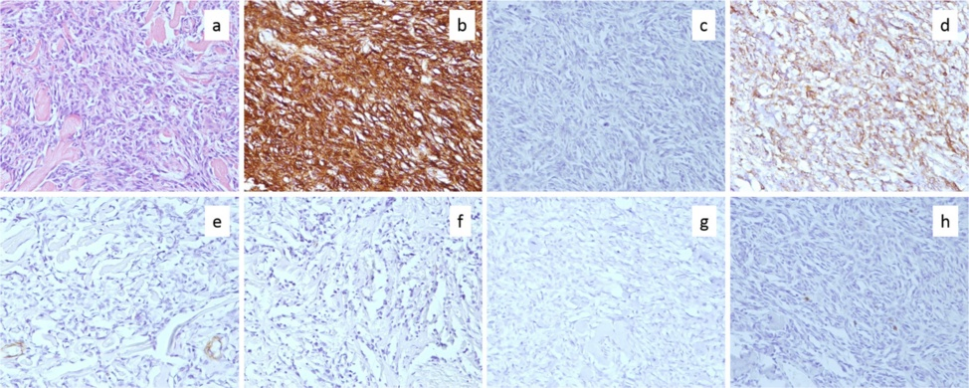
Microscopic findings of the specimen. **a** An image of hematoxylin-eosin staining. Spindle cells with low cytological atypia densely proliferated in much hyalinizing collagen fiber. **b**–**h** Images of immunoreactivity for CD34, Bcl-2, vimentin, SMA, S-100, p53, and Ki-67 are shown, respectively

We performed laparoscopic resection of the mass. We broke up adhesions between the omentum and the right upper abdominal wall and found a white and hard mass in the retroperitoneum over the adhesions (Fig. 2a). It was excised from the retroperitoneum using ultrasonically activated scalpel. Operative duration was 115 min and intraoperative hemorrhage was little. On gross examination, the excised specimen entirely looked like calcification (Fig. 2b). Division surface of the specimen mainly presented calcification and partially red soft tissues (Fig. 2c). Microscopically, small quantities of densely proliferated spindle cells were scattered in major quantities of hyalinizing collagen fiber (Fig. 3a). The spindle cells showed low cytological atypia and no mitosis. Immunohistochemistry showed positive staining of CD34 and vimentin and negative staining of Bcl-2, SMA, A-100, or p53 (Fig. 3b–g). Immunoreaction for Ki-67 was detected in fewer than 1 % of the neoplastic cells (Fig. 3h). The surgical margin was free from the tumor cells. We diagnosed morphologically and immunologically benign SFT in the retroperitoneum. The patient recovered without any postoperative complications, discharged 9 days after the surgery, and had no recurrence 1 year after the surgery.

SFT which is currently known as an uncommon soft tissue neoplasm was first reported as a kind of pleural neoplasms in 1931 [1] and was originally an exclusive diagnosis of pleural neoplasms [2]. Recent electron microscopic and immunohistochemical studies revealed that SFT derives from fibroblastic or myofibroblastic cells under the mesothelium [3, 4]. Thoracic SFT has an age-standardized incidence rate of 1.4 per million, typically occurs in the sixth and seventh decade, and affects both genders equally [5, 6]. Eighty percent of SFT occurs in the thoracic cavity while 20 % of that is found in extrathoracic regions including the retroperitoneum [7, 8]. Retroperitoneal SFT as in the present case is considered to be comparatively rare.

SFT can cause local pressure syndrome [9] and some reports described that SFT presented hypoglycemia due to the insulin-like growth factor II secretion from the tumor [10, 11]. In imaging studies, SFT typically presents well-defined and lobulated mass with well-enhancement [9]. Wignall et al. investigated characteristics of radiologic findings in 34 SFTs and revealed that 62 % of them had the lobulated contours, 9 % had the local invasions, 79 % had heterogenous enhancement patterns, and 65 % had strong contrast enhancement [12]. On magnetic resonance imaging, SFT typically exhibits hypo- or isointensity in T1-weighted image and heterogeneous hyperintensity in T2-weighted image [13], the differential diagnoses of SFT in the retroperitoneum include liposarcoma, leiomyosarcoma, lymphoma, schwannoma, neurofibroma, leiomyoma, myolipoma, angiomyolipoma, sympathetic paraganglioma, desmoid tumor, and epithelioid sarcoma [14]. In the present case, SFT was incidentally detected without any symptoms, imaging inspection provided extremely atypical findings as any retroperitoneal neoplasms including SFT, and thus a precise preoperative diagnosis was difficult. Non-neoplastic lesions such as peritoneal pearl body or foreign body granuloma due to retained surgical sponge in the past appendectomy were exclusively considered, but the imaging findings were not typical of them as well as of neoplastic disease [15]. Fine needle aspiration or biopsy might be difficult due to its marked calcification.

Microscopically, SFT shows multiplying spindle cells in the patternless appearance and the hemangiopericytoma-like appearance with prominent vascularity [16]. England et al. described high cellularity, high mitotic activity (more than 4 mitoses per 10 high-power field), pleomorphism, necrosis, and hemorrhagic changes as the criteria for morphological malignancy of SFT [17]. Those malignant features are observed in about 10 % of SFTs [18]. In immunohistochemistry, CD34 and Bcl-2 are especially useful for differentiating SFT from the other spindle cell neoplasia [9, 19]. Immunoreactivity for CD34 was observed in 98 % and that for Bcl-2 was confirmed in 90 % of SFTs [20]. Other immunological targets such as CD99, desmin, SMA, c-kit, S-100, EMA, CK, CD31, and inhibin are also used for specific diagnosis of SFT [21]. In the present case, the marked calcification was extremely unusual but the scattered tumor cells provided typical structures of SFT with positive immunostaining of CD34 and were considered to be morphologically benign.

The problem regarding malignancy of SFT is discrepancies of morphological malignancy and clinical malignancy [8, 22, 23]. The discrepancy may be partially due to malignant transformation with dedifferentiation of the tumor cells [24, 25]. Therefore, we should not trust morphological findings but immunohistochemistry for some relevant proteins may provide indications of SFT’s clinical malignancy. Takizawa et al. reported that patients with positive immunostaining of both CD34 and Bcl-2 had no recurrence [23]. Loss of immunoreactivity for CD34 and Bcl-2 was observed in the component with malignant transformation [24, 25]. Immunoreaction for p53 was not observed in benign SFT but much confirmed in morphologically and clinically malignant SFT [25]. The present case morphologically yielded typical benign features without immunoreactivity for p53 but can potentially cause a recurrence and should be carefully followed up.

Almost all patients with SFT underwent surgical excision. Many surgical doctors recommend complete surgical excision to obtain clear margins with careful long-term follow-up [8, 9, 16, 26]. There have been few reports concerning chemotherapy for SFT and thus its efficacy is not evident. Baldi et al. used anthracycline and ifosfamide for a patient with recurrent SFT and got partial response; cisplatin and gemcitabine, progressive disease [22]. We performed microscopically complete resection of the tumor and consider follow-up without adjuvant chemotherapy as reasonable.

SFT in the retroperitoneum is rare, and 31 cases have been reported including this case for the last 15 years [9, 14, 16, 18, 20–24, 26–42] (Table 1). The English language literature was extracted from PubMed from 2000 to 2015 using the following Medical Subject Heading terms: “solitary fibrous tumor” and “retroperitoneum”. Age of onset ranged from 17 to 83 and its median was 56. Male to female ratio of the patients was 16:15. Ten patients (38 %) complained of local pressure symptoms in 26 patients with description of any symptoms; 9 patients (35 %), palpable mass; 7 patients (27 %), asymptomatic. Location of the tumor varied all over the retroperitoneal cavity. Size of the tumor ranged from 3.1 to 36 cm and its median was 11.5 cm. No cases were preoperatively diagnosed as SFT. Surgical excision was performed in all cases. Morphologically, 16 cases (62 %) presented benign features while 10 cases (38 %) exhibited malignant findings. Positive immunostaining for CD34, Bcl-2, and p53 was observed at 28 (97 %), 14 (74 %), and 2 (22 %) in described 29, 19, and 9 cases, respectively. Clinical outcomes varied; 21 (78 %) patients remained disease-free after the surgery, 4 patients (15 %) had local recurrence or distant metastasis after the operation, and 3 patients of them died of the disease. One case was diagnosed morphologically benign but suffered distant metastasis 13 years after the surgery and died. The only case of negative immunostaining for CD34 died of the disease. One of the 2 cases in which p53 was immunohistologically positive was dead of the disease. These results indicate that retroperitoneal SFT is quite difficult to diagnose and can be clinically malignant although morphological examinations yield benign findings and negative immunostaining of CD34 or positive immunostaining of p53 can be signs of clinical malignancy.

**Table 1:** Reported 31 cases of solitary fibrous tumor in the retroperitoneum

Reference	Age	Sex	Major complaint	Adjacent structure	Size (cm)	Morphological malignancy	Immunohistochemistry			Outcome
							CD34	Bcl-2	p53	
2008, Takizawa [23]	ND	M	Dysuria	ND	9	Benign	+	+	−	Disease-free
	ND	M	Asymptomatic	Kidney	ND	Benign	+	+	−	Disease-free
2005, Cristi [21]	28	F	Pain	Sacrum	7.5	Benign	+	+	ND	ND
2009, Lau [36]	58	F	Asymptomatic	ND	15	Benign	+	+	ND	7.3 years, disease-free
2011, Charhi [39]	75	M	Palpable mass	Pancreas tail and left adrenal grand	ND	Benign	+	+	ND	ND
2012, Azadi [41]	57	M	Asymptomatic	Pancreas	3.1 and 4.3	Benign	+	+	ND	ND
2014, Toniato [18]	54	M	Severe hypertension	Bilateral adrenal grand	15 and 4	Benign	+	+	ND	1.5 years, disease-free
2004, Kunieda [16]	53	M	Swelling	Right kidney	14	Benign	+	−	−	3 years, disease-free
2015, Maki [The present case]	64	M	Asymptomatic	Liver and right kidney	7.5	Benign	+	−	−	1 year, disease-free
2009, Trabelsi [35]	55	M	Urinary symptom	Bladder	15	Benign	+	−	ND	5 years, disease-free
2000, Morimitsu [26]	72	F	ND	Left kidney	8	Benign	+	ND	−	10 months, disease-free
2001, Clayton [28]	17	F	Hip pain	Spinal column	15	Benign	+	ND	ND	4 years, disease-free
2004, Kume [30]	47	F	Pulsating mass	Superior mesenteric artery	4	Benign	+	ND	ND	1 year, disease-free
2008, Shin [34]	56	M	Asymptomatic	Left external iliac artery and psoas muscle	ND	Benign	+	ND	ND	8 days, discharged
2011, Savas [40]	60	M	Pain	Right kidney	4.5	Benign	+	ND	ND	1 year, disease-free
2013, Baldi [22]	46	F	ND	ND	ND	Benign	ND	ND	ND	13 years, metastasis (dead)
2008, Takizawa [23]	ND	F	Palpable mass	Left kidney	ND	Malignant	+	+	−	Disease-free
	ND	M	Asymptomatic	ND	ND	Malignant	+	+	−	Disease-free
2007, Yamashita [33]	69	F	Swelling	Right kidney	14	Malignant	+	+	ND	2.2 years, disease-free
2011, Bae [38]	59	M	Asymptomatic	Gallbladder	22	Malignant	+	+	ND	3 years, disease-free
2012, Hata [42]	83	M	ND	ND	10	Malignant	+	+	ND	4 years, local recurrence (6 years, alive)
2008, Ito [27]	48	F	Leg edema	Left kidney	5.5	Malignant	+	−	+	2.5 years, disease-free
2009, Nomura [37]	27	F	Palpable mass and pain	Right latissimus dorsi muscle	11	Malignant	+	−	ND	6 months, disease-free
2002, Nakatani [29]	56	F	Palpable mass	Left kidney	9	Malignant	+	ND	ND	2.3 years, disease-free
2015, Kim [9]	52	F	Vaginal discharge	Sacrum	12	Malignant	+	ND	ND	3 years, disease-free
2009, Mosquera [24]	68	F	ND	ND	20	Malignant	−	ND	+	1 month, dead of disease
2004, Nagasako [31]	81	M	Ileus and palpable mass	Pelvis	18	ND	+	+	ND	ND
2005, Yamaguchi [32]	51	F	Pain	Left kidney	10	ND	+	+	ND	ND
2012, Aimé [20]	58	M	Palpable mass	Right kidney	20	ND	+	ND	ND	1 year, disease-free
2013, Nogami [14]	52	F	Emaciation	Pelvis	36	ND	+	ND	ND	Dead on arrival (autopsy case)
2013, Baldi [22]	41	M	ND	ND	ND	ND	ND	ND	ND	23 years, local recurrence (dead)

The case order is sorted by morphological malignancy and findings of immunohistochemistry

*ND* not described

The present case showed prominent calcification in preoperative imaging and the excised mass mostly consisted of calcification with scattered tumor cells. Calcification is not rare for various neoplastic or non-neoplastic lesions in the retroperitoneum such as lipoma, liposarcoma, schwannoma, germ cell tumors, lymphoma after chemotherapy or radiotheraphy, and peritoneal pearl body [43, 44]. Nakashima et al. suggested that the absence of calcification can predict malignancy of primary retroperitoneal tumor [45]. In SFT, calcification was reported to be observed in 3 of 34 cases (8.8 %) [12] and regarded as an indicator of malignancy [46]. However, those kinds of calcification are tiny, scattered, or partial in the lesion. There has been no report of integrally calcified SFT like the present case. The reason why SFT coexisted with such significant calcification is not apparent but the fact is very interesting for considering carcinogenesis or nature of SFT.

## Conclusions

We experienced a case of integrally calcified SFT in the retroperitoneum. There has been no report of SFT with such prominent calcification. Retroperitoneal SFT is difficult to diagnose preoperatively, and careful follow-up after the excision is crucial because morphological malignancy does not necessarily correspond to clinical malignancy.

## Consent

Written informed consent was obtained from the patient for publication of this case report and any accompanying images. A copy of the written consent is available for review by the Editor-in-Chief of this journal.

## Abbreviations

ND: not described
SFT: solitary fibrous tumor
